# The Foreign Journals: Medical Jurisprudence and Toxicology

**Published:** 1840-10

**Authors:** 


### MEDICAL JURISPRUDENCE AND TOXICOLOGY.

*On Poisoning by Antimonial Vapours.* By Dr. Lohmeier, of Schönebeck.

The subjects of this peculiar affection were four in number. The history of the first is as follows:

D. W. E. F. was affected in 1838 with tightness in the chest and slight pain of the head. The former symptom increased to pain, and at last to severe stitches passing transversely through the chest to the shoulders and the back, to which there was also added a severe dry and painful cough. With these symptoms the pain of the head, which was at first but slight, acquired an agonizing severity, with stabbing and burning sensations especially at the back of the head and the neck, and with swelling of the cervical glands. The dry painful cough at last produced a difficult expectoration, while in respiration a rhonchus and sibilus were constantly audible. At night a tormenting restlessness kept the patient awake; and this soon passed into complete watchfulness; or, if he fortunately had a short sleep, he fell into a distressing colliquative sweat which was followed by great faintness. The appetite diminished from the very commencement of the illness, and diarrhœa came on with griping pains in the abdomen. The diarrhœa was troublesome and frequent; the food was evacuated undigested soon after eating; and the abdomen was enlarged and tense. There were pain and difficulty in making water, and at last the passage of the urine was always accompanied by strangury and pain at the neck of the bladder, and burning sensations in the urethra, from which a few drops of a liquid mucus sometimes flowed. Pains in the testicles were felt, and there was a loss of the desire for the exercise of the sexual propensity, which went on to actual impotence; and the patient observed a remarkable shrinking of the penis and an actual diminution in the size of the testicles.

The cases of the three other patients are so nearly similar to the above that they need not be detailed. After describing them, Dr. Lohmeier proceeds: These histories had so much similarity that one and the same cause of disease in all the patients might fairly be anticipated; and it was the opinion of one of them who, like the others and some more who they said were similarly affected, had been occupied at the time of his illness in making antimonial preparations on a large scale, that the vapours given off during the process were the cause of his disorder. The idea, however, was not followed out till the same patient having almost entirely recovered from his first attack, but being still annoyed by pains, found them suddenly break out again into more severe symptoms on being exposed to the vapours developed in preparing a large quantity of tartar-emetic.

The author was now induced to enquire more accurately into the origin of these affections, which both by their history and their symptoms appeared so strongly indicative of the influence of some irritating poison. He found that one of the patients, D. W. F., at the time of his illness had been engaged for fourteen days successively in the manufacture of metallic antimony, by melting algorath-powder with fluxes composed of salts of potash and soda, at first in crucibles and afterwards in a small blast furnace. He melted every day about a hundredweight of metal, which was done in from four to six meltings, according to circumstances; and he had to test it during the time, and looked after the stirring of it himself. During this process vapours of antimonious and antimonic acids, and of oxyde of antimony are given off, from those substances being mixed with each quantity of the algaroth-powder that is thrown into the furnace.

G. H., another of the patients, had the management of solutions of antimony in hydrochloric acid in an open cast-iron cauldron, in which vapours of hydrochlorate of antimony (butter of antimony) were formed.

J. F., the third patient, was engaged in the same work with the last; and K. J., the fourth, was constantly occupied in preparing first the *vitrum antimonii*, and afterwards the artificial sulphuret, which is made by melting together sulphur and metallic antimony; in both of which processes vapours are given off containing antimonious and antimonic acids and oxyde of antimony.

There could, therefore, be no doubt that the peculiar and severe symptoms produced in these cases were the result of the absorption of the different compounds of antimony in the form of vapour. The remainder of the very interesting paper is occupied by the author's speculations respecting their mode of reception into, and action upon, the system; and by his suggestions for the prevention and the cure of the injurious effects to which they give rise. For the latter purpose he recommends local bleedings from the neighbourhood of the parts chiefly affected, and the concurrent administration of tonics, especially bark, which he presumes must exercise a chemical influence on the poison. For the prevention of poisoning no other than the usual means, such as powerful draughts of air for the removal of the vapours as fast as they are given off, seem to be necessary.

*Casper's Wochenschrift. April und Mai,* 1840.

